# Effects of Four Host Plants on Biology and Food Utilization of the Cutworm, *Spodoptera litura*

**DOI:** 10.1673/031.010.2201

**Published:** 2010-03-23

**Authors:** Ming Xue, Yun-Hong Pang, Hong-Tao Wang, Qing-Liang Li, Tong-Xian Liu

**Affiliations:** ^1^ College of Plant Protection, Shandong Agricultural University, Taian, Shandong 271018, China; ^2^ Department of Entomology, Texas AgriLife Research, Texas A&M University System, Weslaco, Texas 78596-8399, USA; ^3^ Key Laboratory of Applied Entomology, Northwest A&F University, Yangling, Shaanxi 712100, China

**Keywords:** development, food utilization, longevity, oviposition, survival, Chinese cabbage, cowpea, sweet potato, tobacco

## Abstract

Effects of four host plants, tobacco, Chinese cabbage, cowpea and sweet potato, on larval and pupal development and survival, and longevity and fecundity of adults of *Spodoptera litura* (F) (Lepidoptera: Noctuidae), were studied under laboratory conditions (26° C, 60–80% RH), as was the utilization of the four host plants and adaptation on tobacco. All of the biological parameters included in the study were affected by the host plants. In a choice test, *S. litura* females oviposited most on Chinese cabbage, least on tobacco, and intermediate on cowpea and sweet potato. *S. litura* larvae developed differently on the four host plants, from shortest to longest in the following order: Chinese cabbage, cowpea, sweet potato, and tobacco. Pupal development was shorter on cowpea than on the other three host plants, and males generally developed longer than females. More females than males were found among emerged adults, and male adults lived 1–2 d longer than females. Larvae survived best on cowpea (81.6%), followed by Chinese cabbage (75.5%), then sweet potato (66.1%), and worst on tobacco (49.2%). Pupal survival rates were relatively high (91.4 – 95.9%) in all four host plant treatments, although that on sweet potato was lower than those on the other three host plants. Pupal weights on tobacco and sweet potato were similar, but both were lower than those on Chinese cabbage and cowpea. Generally, male pupae weighed less than female pupae. Numbers of eggs oviposited by female *S. litura* were highest on sweet potato, followed by those on cowpea, Chinese cabbage, and lowest on tobacco. Relative food consumption rate was highest on sweet potato, followed by that on cowpea, Chinese cabbage, and lowest on tobacco. In contrast, *S. litura* larvae that fed on tobacco had higher efficiency of conversion of digested food, highest efficiency of conversion of ingested food, and lowest approximate digestibility as compared with larvae that fed on other host plants. The potential causes for *S. litura* outbreaks on tobacco are discussed.

## Introduction

*Spodoptera litura* (Fabricius) (Lepidoptera: Noctuidae) is polyphytophagous, damaging numerous vegetables and field crops in China and many other Asian countries (Shu 1959; Hill 1975; Shivayogeshwara 1991). *S. litura* is also known as the common or tobacco cutworm, or the cluster or tobacco caterpillar. Although it had been a sporadic pest of tobacco in northern China for many years, it has been becoming gradually a very important insect pest in recent years (Guan and Chen 1999; Gao et al. 2004; Qin et al. 2004). It also becomes resistant to many commonly used insecticides, particularly pyrethroids and carbamates, resulting in failure of effective controls (Wu et al. 1995; Kranthi et al. 2002; Ahmad et al. 2007; Huang and Han 2007).

Study of the effects of host plants on the biology of insects is important in understanding host suitability of plant-infesting insect species. There have been a number of studies on the biological parameters of *S. litura* on different host plants under different environmental conditions, particularly in India (Patel et al. 1986, 1987), Pakistan (Ahmad et al. 2007), China (Guan and Chen 1999; Zhu et al. 2000; Qin et al. 2004; Zhu et al. 2005), Korea (Bae et al. 1997; Bae 1999a,b; Bae and Park 1999), and other Asian countries (Etman and Hooper 1979; Holloway 1989) where *S. litura* has been an important pest on various crops. However, not all of these studied the effects of the same host plants on development, survival, pupal weight and oviposition of *S. litura* under the same environmental conditions, and none studied the adaptation of *S. litura* on tobacco for two generations after they were reared on an artificial diet.

Thus, the objectives of this study were to determine (a) oviposition preference by the females and the development and survival of larval and pupal stages of *S. litura* on tobacco, Chinese cabbage, cowpea and sweet potato and (b) food utilization on the four host plants.

## Materials and Methods

### Host plants

Four host plants were used in this study, including tobacco (*Nicotiana tabacum* L., variety ‘NC89’), Chinese cabbage (*Brassica rapa* var. *chinensis*, variety ‘Shandong Fushen Baotou’), cowpea (*Vigna sinensis* L. Walp. ssp. *uniguiculata*, variety ‘Zhijiang 28-2’), and sweet potato (*Ipomoea batatas* L., variety ‘Yushu 10’). These plants were selected because they are the most important economic crops in northern China and are primary host plants of *S. litura*.

The four species of host plants were singly planted in plastic pots (19-cm in diameter and 14 cm in depth) in a greenhouse at Shandong Agricultural University at Taian, Shandong, China, and were maintained insecticide-free. The plants were used when they had 4–5 true leaves.

### Insects

*S. litura* larvae were originally collected from cabbage fields (*Brassica oleracea* var. *capitata* L.), and were subsequently reared on an artificial diet at the Institute of Pomology, Guangdong Academy of Agricultural Science, Guangzhou, Guangdong. The artificial diet contained the following ingredients: 137 g of corn flour, 10 g of yeast, 37.5 g of soybean flour, 3.5 g of multiple vitamins, 1 g of sorbic acid, 2 g of nipagin, 12.5 g of agar, 0.16 g of inositol, and 0.15 g cholesterol. Soybean flour and corn flour were sterilized for 40 min before they were used. The diet was prepared as described by Zhu et al. (2001).

### Host preference for oviposition

This experiment was conducted in an airconditioned insectary at 26 ± 1° C, 12:12 L:D, and ≈70% RH. *S. litura* adults were developed from the larvae that had been fed with the artificial diet for three generations. Newly emerged adults were collected and released in each cage at a sex ratio of 1:1. The adults were fed with 10% sugar-water solution through a cotton ball in a small plastic container (3.5× 1.3 cm). The adults were allowed to mate in the cage for two days. Eight plants were placed in a screen cage (50×50×50 cm), and two plants from each species were randomly placed at one of the four corners. The plants were adjusted to the same height. On the third day, 10 pairs of adults (5 females and 5 males) were released in each screen cage containing eight plants, and again, 10% sugar-water solution was supplied for the adults. The females were allowed to oviposit for 2 days, and the number of egg masses and eggs in each egg mass was recorded for each plant. The experiment was replicated six times.

### Larval development and adult reproduction

Newly hatched larvae reared on the artificial diet were transferred and reared separately in small containers (3.5×1.3 cm) on each of the four host plants until they reached the fifth instar. Newly exuviated fifth instars were individually reared in Petri dishes (9.0 × 1.5 cm) to avoid cannibalism. Each host plant treatment had 100 larvae. The larvae were monitored for development and mortality at 12-h intervals. In the meantime, the dishes were cleaned, and new leaf pieces were replaced as needed. Before pupation, a few pieces of paper tissue were placed on the dish bottom for larval pupation. Two-day old pupae were sexed and separately weighed. Newly emerged adults were placed in a container (6.5 × 12 cm) and were fed with 10% sugar-water solution. The females would readily oviposit on paper stripes (1 cm wide, 5–10 cm long) in the container. The adults were monitored daily for mortality and oviposition, and number of egg masses oviposited on the paper stripes by each female were collected and counted twice daily until the female died.

### Food consumption and utilization

Newly exuviated sixth instar larvae that had been reared on each of the four host plant species for two generations were used in this study. Larvae of approximately the same size were selected and individually placed in small containers. The larvae were starved for 10 h, and then each larva was individually coded and weighed. Twenty larvae were used in each of the four host plant treatments. The larvae were equally divided into a control group and a treatment group. In the control group, the 10 larvae and 10 fresh leaves from each of the four host plants were individually weighed, dried in a drier at 80° C, and weighed again. The dry weights were used as the standard for all other treatments. In the treatment group, leaves detached from each of the four host plants were weighed and provided to the 10 larvae. The larvae were fed with the leaves for 48 h. The larvae were then starved for 6 h to allow the larvae to defecate. The larvae, leaf tissues, and feces in each dish were weighed and then dried in a drier at 80° C. The dried leaf tissues and larvae were weighed again. Food utilization rates were then calculated based on the formulas of Waldbauer (1968):

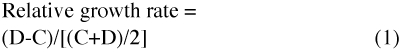


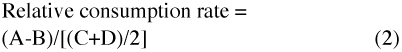


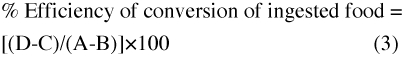


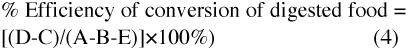


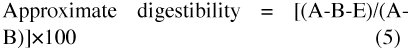

Where A is the weight of dried leaf tissues in the control, B is the weight of the dried leaf tissue in each treatment, C is the weight of dried larvae in the control, D is the weight of dried larvae in each treatment, and E is the weight of dried feces in each treatment.

### Data analysis

The life history parameters of *S. litura* were analyzed using one-way and factorial ANOVA (SAS Institute 2008). Means associated with host plants for each variable were separated using the least significant difference test when significant values were obtained.

## Results

### Host plant preference for oviposition

**Figure 1.**
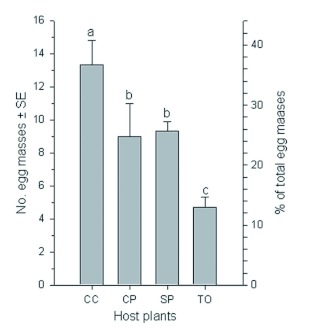
Egg masses per female *Spodoptera litura* on four host plants. Host plants: CC - Chinese cabbage, CP cowpea, SP - sweet potato, and TO - tobacco. The same letters over the four bars in each figure indicate that the means are not significantly different at p < 0.05 (Least Significance Difference test, SAS Institute 2008). High quality figures are available online.

In the choice test, numbers of egg masses oviposited by *S. litura* females on the four host plants differed significantly (*F* = 17.73; df = 3, 23; p = 0.002) (Figure 1). *S. litura* oviposited the most on Chinese cabbage (13.3 egg masses, 36.7%), followed by sweet potato (9.3 egg masses, 26.7%) and cowpea (9.0 egg masses, 24.8%), and the least on tobacco (4.7 egg masses, 12.8%).

### Larval and pupal development

**Figure 2.**
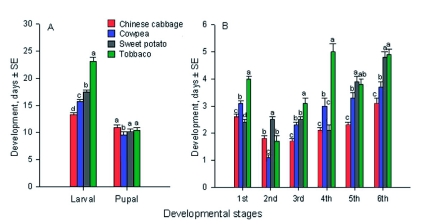
Development of *Spodoptera litura* larvae on four host plants. Host plants: CC - Chinese cabbage, CP cowpea, SP - sweet potato, and TO - tobacco. The same letters over the paired-bars in each figure indicate that the means are not significantly different at p < 0.05 (Least Significance Difference test, SAS Institute 2008). High quality figures are available online.

Overall larval development was significantly affected by host plants (*F* = 26.26; df = 3, 15; p < 0.0001) (Figure 2), and was longest on tobacco (23.2 d), followed by sweet potato (17.5 d), cowpea (15.8 d), and shortest on Chinese cabbage (13.3 d) (Figure 2A). Of the six instars, the first, third and fourth instars development took significantly longer on tobacco (*F* = 58.01 - 91.68; df = 3, 15; p < 0.0001) (Figure 2B). In contrast, second instar development took significantly longer on sweet potato than on the other three host plants, and the for the fifth and sixth instars development time was not significantly different for tobacco and sweet potato (Figure 2B). Pupal development times on Chinese cabbage (10.9 d), cowpea (10.1 d) and sweet potato (10.1 d) were not significantly different, and were longer than on cowpea (9.5 d) (*F* = 6.84; df = 3, 15; p < 0.05) (Figure 2A).

### Larval and pupal survival

**Figure 3.**
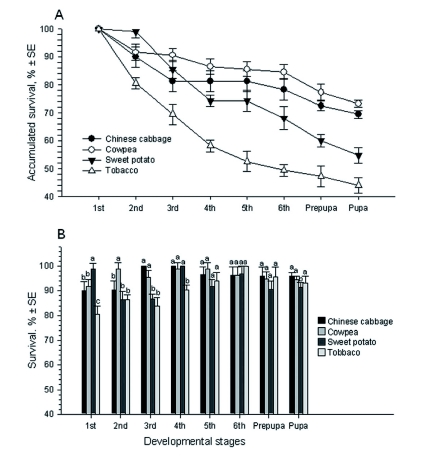
Accumulated survivals (A) and stage-specific survivals (B) of *Spodoptera litura* on four host plants. Host plants: CC - Chinese cabbage, CP - cowpea, SP - sweet potato, and TO - tobacco. The same letters over the four bars in each figure indicate that the means are not significantly different at p < 0.05 (Least Significance Difference test, SAS Institute 2008). High quality figures are available online.

The survival rates of *S. litura* larvae varied on the four host plants (Figure 3). The overall, accumulated survival rates of all larval stages on the four host plants differed significantly (*F* = 26.43; df = 3, 15; p < 0.05) (Figure 3A) and was lowest on tobacco (49.0%), followed by that on sweet potato (66.2%), on Chinese cabbage (75.4%), and highest on cowpea (81.7%). Of the six larval stages, the survival rates of *S. litura* were significantly different in the first four instars (*F* = 4.03 - 42.27; df = 3, 15; p = 0.0339 - 0.0001), but not in the two oldest ones (*F* = 0.83 and 2.62; df = 3, 15; p = 0.0987 and 0.5018) (Figure 3B). Different larval instars responded differently on each of the four host plants. The survival rates of the first instar were highest on sweet potato (99.0%) and lowest on tobacco (80.1%), with intermediates on Chinese cabbage (90.0%) and cowpea (91.7%). The survival rates of the second instar were not significantly different on Chinese cabbage (90.3%), sweet potato (86.5%) and tobacco (80.6%), but were significantly higher on cowpea (98.9%) than on the other three host plants. The survival rates of the third instars were similar on Chinese cabbage (100%) and cowpea (95.5%), which were higher than those on sweet potato (86.8%) and tobacco (83.8%). The fourth instars survived less on tobacco (90.3%) than on the other three host plants (98.6 – 100%). The survival rates for prepupae were not significantly different (*F* = 0.93; df = 3, 15; p > 0.05), but those for pupae were (*F* = 4.56; df = 3, 15; p < 0.05).

**Table 1.**
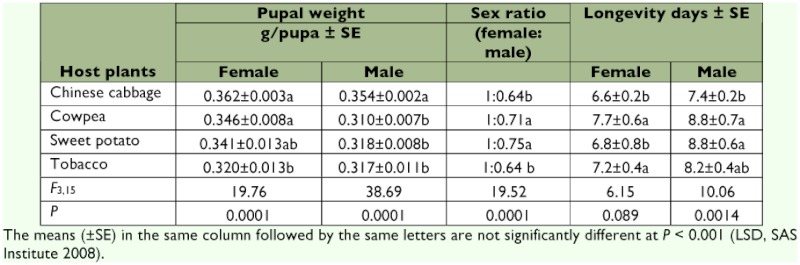
Effects of four host plants on pupae and adults of *Spodoptera litura*.

### Pupal weight

Pupal weights differed significantly depending on the host plants on which the larvae were fed and differed significantly between females and males when they fed on the same host plants and when larvae fed on different host plants (p < 0.005) (Table 1). The female pupae on Chinese cabbage were heaviest, followed by those on cowpea and sweet potato, and lightest on tobacco, and the male pupae on Chinese cabbage were heavier than on the other three host plants. Female pupae were generally heavier than their male counterparts, except for those on tobacco, which were the same weight.

**Figure 4.**
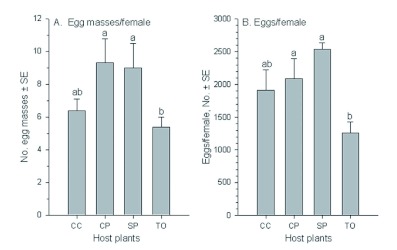
Egg masses and total eggs per female *Spodoptera litura* on four host plants. Host plants: CC - Chinese cabbage, CP - cowpea, SP - sweet potato, and TO - tobacco. The same letters over the four bars in each figure indicate that the means are not significantly different at p < 0.05 (Least Significance Difference test, SAS Institute 2008). High quality figures are available online.

### Sex ratio, adult longevity and oviposition

Sex ratios were biased, and more female adults emerged than male adults when their larvae were fed with the four host plants (*F* = 19.52; df = 3, 15; p = 0.0001) (Table 1). Of the six treatments, male ratios were higher on cowpea and sweet potato than on the other two host plants. The longevities of both female and male *S. litura* adults were also significantly affected by the host plants on which their larvae fed (female: *F* = 6.15; df = 3, 15; p = 0.0089; male: *F* = 10.6; df = 3, 15; p = 0.0014). Numbers of egg masses and total eggs oviposited by *S. litura* females on the four host plants differed significantly (egg masses: *F* = 16.61; df = 3, 15; p = 0.0001; total eggs: *F* = 31.13; df = 3, 15; p = 0.0001) (Figure 4). *S. litura* oviposited similar numbers of egg masses and eggs per female on cowpea, sweet potato, and Chinese cabbage, but less on tobacco than on the other three host plants. Numbers of eggs per egg mass were similar on Chinese cabbage (283.5 eggs/egg mass) and sweet potato (282.1 eggs/egg mass), which were more than those on cowpea (224.9 eggs/egg mass) and tobacco (233.6 eggs/egg mass).

### Food consumption and utilization

Food consumption and conversions of ingested and digested food by *S. litura* larvae varied considerably among the four host plants that the larvae consumed (Table 2). The relative growth rates on tobacco (0.43), cowpea (0.43) and Chinese cabbage (0.40) were higher than that on sweet potato (0.32) (*F* = 8.050; df = 3, 39; p < 0.001). The relative consumption rates were highest when the larvae fed on sweet potato (3.90), followed by that on cowpea (3.16), then on Chinese cabbage (2.28), and the lowest on tobacco (1.51) (*F* = 56.19; df = 3, 39; p < 0.001). The efficiency of conversion of ingested food was highest on tobacco (29.75), followed by that on Chinese cabbage (17.85), then that on cowpea (14.04), and lowest on sweet potato (8.34) (*F* = 74.59; df = 3, 39; p < 0.001). The efficiency of conversion of digested food was higher when the larvae fed on tobacco than when fed on the other three host plants (*F* = 18.73; df = 3, 39; p < 0.001).. However, the larvae that fed on Chinese cabbage and cowpea were similar and lower than those on sweet potato and tobacco. The approximate digestibility of *S. litura* larvae on the four host plants differed significantly (*F* = 63.56; df = 3, 39; p < 0.001) and were higher on Chinese cabbage and cowpea than on sweet potato and tobacco.

**Table 2.**
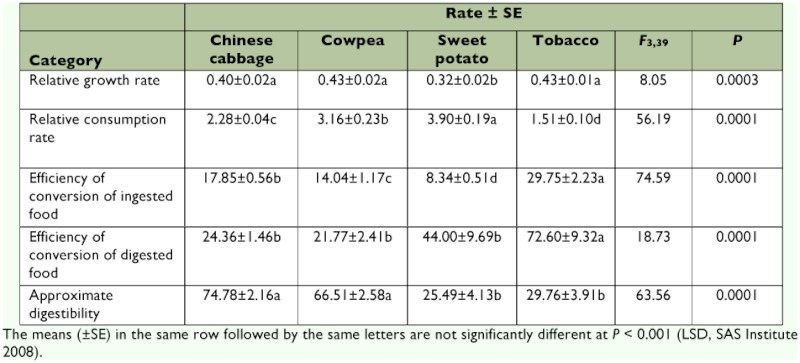
Nutritional indices of *Spodoptera litura* larvae feeding on four host plants.

## Discussion

The data clearly show that *S. litura* performed differently in oviposition, larval and pupal development and survival, pupal weight, and oviposition of emerged females when Chinese cabbage, cowpea, sweet potato and tobacco were offered as the food plants for their larvae. These results are supported by a number of studies, although direct comparison of these data can be difficult because different host plants and environmental conditions were used in these studies. Although the same insect was used, it differed in origins, and it could be different strains or biotypes. However, in a few studies, the same host plants (one or two) were used, including cowpea (Qin et al. 2004; Zhu et al. 2005) and Chinese cabbage (Zhang et al. 1997). Qin et al. (2004) used the same variety of cowpea but found that the developmental time of *S. litura* larvae was 12.8 d at 29° C, which was 3 d shorter than in this study. Zhu et al (2005) found that *S. litura* larvae finished their development in 10.1 d on cowpea as compared with 15.8 d in the current study. This difference (5.7 d) could be caused by higher temperature (28.1° C) or different cowpea variety from what was used here (26° C; ‘Zhijinag 28-2’).

Larval development of *S. litura* varied greatly depending on host plants and temperature, and the development was prolonged under low or high temperatures (Zhu et al. 2000; Chen et al. 2002; Seema et al. 2004). For example, on tobacco, larval development can range from 19.3 d at 26° C (Chen et al. 2002), 23.2 d in this study at 26° C, to as long as 30 d at 23° C (Rattan and Nayak 1963). Similarly, on cowpea, larval development ranged from 10.1 d at 28° C (Zhu et al. 2005) to 15.8 d at 26° C in the present study.

It has been reported that pupal development was not affected by host plants on which their larvae fed (Patel et al. 1986). However, our results show that pupae developed faster on cowpea than on Chinese cabbage, sweet potato and tobacco, although the difference was <1 d longer on sweet potato, 1 d longer on tobacco, and 1.5 d longer on Chinese cabbage. Bae and Park et al. (1999) found that temperature plays a vital role on pupal development. Bae and Park (1999) reported that the mean pupal developmental duration was as long as 13.8 d at 24° C on perilla to as short as 7.4 d at 32° C on soybean.

Larval survival or pupation rate of *S. litura* varied greatly on different host plants, ranging from 100% on *Ricinus communis* (Patel et al. 1987) to 49.0% on tobacco in this study. Bae and Park (1999) found that pupation rates were positively correlated with high temperature, 30.0, 33.3 and 38.5% on sweet potato, perilla and soybean, respectively, at 24° C, and 57.5, 80.0 and 87.5% at 32° C on the same host plants, respectively.

These results show that larval food directly affects pupal size and weight, and the female pupae were heavier than male pupae on all four host plants (Table 1). The pupal weights of *S. litura* in this study were 0.32 to 0.36 g, which were generally within a wide range on various host plants, from 0.28 g on sweet potato to 0.40 g on perilla and cowpea (Bae and Park 1999; Qin et al. 2004). Pupal weights were not found to be significantly different among different temperature regimes (Seema et al. 2004), but 27° C was considered the optimum temperature for larval and pupal growth and development. Bae and Park (1999) found that pupal weight tended to be 3 – 13% lower with increasing temperature from 24° C, to 28° C, then to 32° C.

In the present data, more than 91% of *S. litura* pupae successfully developed to adults. In contrast, Bae and Park (1999) reported significantly low emergence rates on four foods. They found that only 31.4% of pupae developed to adults at 24° C and 88.6% at 30° C, and their emergence rate increased 1.1 to 2.8 fold with temperature increase from 24° C to 32° C.

Numbers of eggs of *S. litura* oviposited by the females from the larvae that fed on the four host plants were generally within the range as reported on various host plants (Figure 3). In the literature, oviposition by females varied greatly on different hosts under different environmental conditions (Patel et al. 1986; Bae and Park 1999). Numbers of eggs laid by a single female ranged from 935 on soybean (Bae and Park 1999) to 3,467 on cotton (Patel et al. 1986). In contrast, Bae and Park (1999) reported that *S. litura* females oviposited an average of 803 eggs on an artificial diet, and Chu and Yang (1991) found that *S. litura* oviposited as many as 5,995 per female on a different artificial diet.

In the present study, male adults generally lived longer (7.4 – 8.8 d) than females (6.6 – 7.7 d), differing on different host plants (Table 1). Similar results were reported by Bae and Park (1999), although the differences were generally less than 1 d (0.25 – 1.0 d). However, Patel et al. (1986) found that on cotton, male adults lived 6.3 d as compared with 12.3 d for female adults. It has been found that adult longevity became shorter as the temperature increased (Bae and Park 1999). In the present study, the sex ratio was biased, and more females emerged than males with sex ratios of 1.0:0.64 – 0.75. This was supported by Seema et al. (2004). Differences in pupal survival, adult sex ratio, longevity, and fecundity may also be affected by temperature and other environmental conditions (Zhu et al. 2000; Chen et al. 2002; Seema et al. 2004).

Our data show that all nutritional indices varied when *S. litura* fed on the four host plants. Food conversion efficiencies on different host plants vary considerably by *S. litura* larvae (Balasubramanian et al. 1985) and by insects in general (Scriber and Slansky 1991; Slansky and Scriber 1985). The current data show that *S. litura* had similar relative growth rates on Chinese cabbage, cowpea, and tobacco, but had the lowest relative consumption rate when feeding on tobacco compared with those for the other three host plants. However, the larvae were more efficiently converting tobacco tissues into their biomass than other plant tissues as shown by larvae fed on tobacco having the lowest approximate digestiblity and the highest efficiency of conversion of digested and ingested food. One cause of such variation may involve homeostatic adjustment of consumption rates and efficiency parameters such that an insect can approach its “ideal” growth rate even with foods of different quality. Zhu et al. (2005) found that *S. litura* larvae did not prefer feeding on banana leaves and had lower relative growth rate, relative consumption rate, and approximate digestibility, but it had a significantly higher efficiency of conversion of ingested food and an extremely higher rate of efficiency of conversion of digested food, indicating that the larvae are capable of compensating by more efficiently utilizing their limited banana leaf tissues than other host plants. The digestion rate is affected by the enzyme activities of various host plants, including trehalase, invertase, and others. In practice, however, it can be quite difficult to ascertain “cause” and “effect” responses with efficiency parameters. Does the insect eat more because digestibility is low, or is digestibility low because the insect is eating more? Efficiency parameters are so closely related physiologically that determination of “cause” and “effect” is not a trivial matter. Factors contributing to such changes are still largely unknown, but may include shifts in food selection, digestive physiology, metabolic rates, and body composition. Understanding of these basic principles of nutritional ecology can enhance our appreciation of insects' adaptation to new food resources.

As this study found that *S. litura* dose not prefer feeding on tobacco, why has it been able to cause severe damage on tobacco in China? Our data indicate that *S. litura* was able to adapt on tobacco in a relatively short period of time (Xue, unpublished data). For example, comparison of the development, survival, and fecundity parameters between the two generations clearly show that the larvae in the second generation developed faster, survived more, had heavier pupae, and oviposited more. These results may partially explain the facts that *S. litura* could adapt on tobacco and quickly become a severe pest. The outbreaks of *S. litura* can be affected by many biotic and abiotic factors. Gao et al. (2004) identified several factors that may cause outbreaks of *S. litura* in northern China. Those factors include increasing acreage of tobacco and many preferred crops (mainly vegetables), which provides abundant food sources; expanding of protected cultivations, which provides suitable sites for overwintering; more mild winter and warmer spring, which enable the pest to occur earlier and build up higher spring populations in the first generation; high temperature and less rainfall in summer; and misuse of pesticides that cause resistance to insecticides and less natural enemies. Monoculture could be another factor because it is more favorable for the pest, and irrigation may also contribute to the outbreaks of *S. litura* in China. The occurrence of *S. litura* is generally synchronous with the rapid growing period of tobacco, especially during summer, providing plenty of sources for oviposition and larval feeding. In India, Pandey (1977) considered a good rainfall after a long period of drought as the key factor causing *S. litura* outbreaks. *S. litura* have also become resistant to most commonly used insecticides (Wu et al. 1995; Kranthi et al. 2002; Huang and Han 2007). In addition, culture practices, cropping systems, irrigation, and other weather conditions (i.e., typhoon) affect the outbreaks of *S. litura* on various cropping systems around the world (Murata et al. 1998; Chaudhary and Bajpai 2006).

In conclusion, based on oviposition preference, larval development, survival, growth, pupal weight and duration, and emergence and fecundity of adults of *S. litura*, the preference and nutritional values of the four host plants were ranked as Chinese cabbage > cowpea > sweet potato > tobacco. Hence, the present study has shown the suitability of selected host plants for the development, longevity, and survival of *S. litura.* These findings will help to understand the biology of this particular pest and could help in its management and control, particularly on tobacco. Therefore, future studies should focus on testing a wider range of host plant species for the development of *S. litura*, and assessment of the chemical components of the host plant species would help to better understand the mechanism of host suitability.
